# Biphasic Myopathic Phenotype of Mouse DUX, an ORF within Conserved FSHD-Related Repeats

**DOI:** 10.1371/journal.pone.0007003

**Published:** 2009-09-16

**Authors:** Darko Bosnakovski, Randy S. Daughters, Zhaohui Xu, Jonathan M. W. Slack, Michael Kyba

**Affiliations:** 1 Lillehei Heart Institute and Department of Pediatrics, University of Minnesota, Minneapolis, Minnesota, United States of America; 2 Faculty of Technology and Technical Science, University St. Kliment Ohridski, Veles, Republic of Macedonia; 3 Stem Cell Institute, University of Minnesota, Minneapolis, Minnesota, United States of America; 4 Department of Developmental Biology, University of Texas Southwestern Medical Center, Dallas, Texas, United States of America; Brunel University, United Kingdom

## Abstract

Facioscapulohumeral muscular dystrophy (FSHD) is caused by contractions of D4Z4 repeats at 4q35.2 thought to induce misregulation of nearby genes, one of which, DUX4, is actually localized within each repeat. A conserved ORF (mDUX), embedded within D4Z4-like repeats, encoding a double-homeodomain protein, was recently identified on mouse chromosome 10. We show here that high level mDUX expression induces myoblast death, while low non-toxic levels block myogenic differentiation by down-regulating MyoD and Myf5. Toxicity and MyoD/Myf5 expression changes were competitively reversed by overexpression of Pax3 or Pax7, implying mechanistic similarities with the anti-myogenic activity of human DUX4. We tested the effect of mDUX expression on *Xenopus* development, and found that global overexpression led to abnormalities in gastrulation. When targeted unilaterally into blastomeres fated to become tail muscle in 16-cell embryos, mDUX caused markedly reduced tail myogenesis on the injected side. These novel cell and animal models highlight the myopathic nature of sequences within the FSHD-related repeat array.

## Introduction

Facioscapulohumeral muscular dystrophy (FSHD), one of the most common inherited myopathies, is caused by a contraction within a subtelomeric array of D4Z4 repeats 4q35.2. FSHD-affected individuals carry from 1 to a maximum of 10 repeats, while unaffected individuals have from 11 to 150 copies of 3.3 kb D4Z4 elements [1], [2]. The D4Z4 repeat is very GC rich and contains an intronless double homeobox gene named DUX4 (double homeobox, chromosome 4). It also contains a heterochromatic LSau sequence and sequence elements that bind the repressor elements YY1, HMGB2 and nucleolin [3]. Several molecular mechanisms have been proposed to link the D4Z4 contraction to up-regulation of FSHD candidate genes, including multimerization of YY-1-containing transcriptional repressor complexes [2], hypomethylation of the contracted D4Z4 allele [4], differential looping interactions [5] or loss of a barrier to an enhancer distal to the repeats [6].

A C-terminally-truncated inverted D4Z4 element is localized 42 kb centromeric to the array and referred as DUX4c [7]. A number of other FSHD candidate genes, including FRG1, TUBB4Q, FRG2 and ANT1 [2], [8], [9], [10] are also localized to the centromeric side of the D4Z4 array. Recent publications have documented low but detectable levels of DUX4 transcript and protein in biopsy samples and myoblast cultures from FSHD patients but not unaffected controls [11], [12]. We have recently shown that DUX4, but not DUX4c, increases susceptibility to oxidative stress due to downregulation of the genes involved in the glutathione redox cycle, and that both DUX4 and DUX4c rapidly downregulate MyoD expression resulting in impaired myogenic differentiation [13], [14]. Both changes in glutathione redox potential and effects on MyoD have been identified in studies on FSHD myoblasts and biopsies [15], [16], [17], [18], [19]. We also demonstrated that the key myogenic regulators Pax3 and Pax7, two proteins with homeodomains similar to DUX4 and predicted to compete for the same DNA recognition sequences, did in fact compete for regulation of MyoD and Myf5, and significantly reversed the toxicity of DUX4 to myoblasts [14].

Recently, Clapp et. al. analyzed DNA sequence data from primates, rodents, afrotherian and other species and concluded that a D4Z4-like repeat family containing an ORF encoding a double homeodomain, is evolutionally conserved and arose over 100 million years ago [20]. The mouse representative (named mDUX) contains a 2 kb ORF embedded within a 5 kb repeat unit on chromosome 10 [20]. Using specific partial primer sets and *in situ* hybridization, bidirectional transcription from different tissues including brain, heart, lung and muscle was documented. The possibility of bidirectional transcription, (as well as various splice forms, and potential miRNAs within D4Z4) has also been demonstrated by Snider et al. [21].

Although the double homeodomain sequences are strongly conserved between mDUX and human DUX4, the remainder of the proteins are highly divergent. We therefore tested whether mDUX expression could affect viability, myogenic gene expression, or myogenic differentiation potential both *in vitro* using a cell-based assay, and *in vivo* by microinjection of mDUX RNA into developing *Xenopus* embryos. We find remarkable molecular similarities between mouse DUX and human DUX4 or DUX4c and discuss the value of engendering animal models based on altering the regulation of endogenous D4Z4-like repeats, or conditionally expressing of the ORF they encode.

## Results and Discussion

### Generation of mDUX inducible cell lines and evaluating mDUX toxicity

We wished to use a conditional gain of function approach to analyze the effect of mDUX, because we reasoned that mDUX could have toxic characteristics similar to human DUX4 [13], [14]. For this purpose, we used C2C12 myoblasts, 3T3 fibroblasts, and murine ES cells modified for inducible cassette exchange, a method that enables rapid gene targeting to a locus that is tightly and conditionally regulated. In these cell lines, mDUX can be turned on and off, and expression can be regulated over a 3-log range of mRNA levels by titrating the concentration of the inducer, doxycycline [14]. We refer to the derivative inducible cell lines as iC2C12-mDUX, i3T3-mDUX and iES-mDUX, respectively.

**Figure 1:**
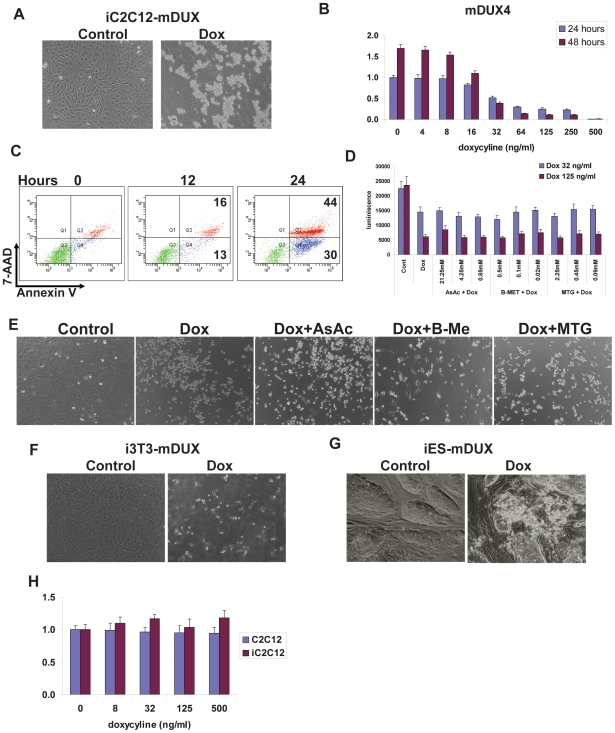
Toxicity of mDUX. (A) Morphology of iC2C12-mDUX cells induced for 24 hours (Dox) with 500 ng/ml doxycycline. The majority of induced cells were detached and floating after 24 hours. (B) ATP assay for analysis of viability in iC2C12-mDUX cells induced with various concentrations of doxycyline for 24 and 48 hours. Decreased cell viability was significant in the cells induced with as little as 32 ng/ml doxycycline in the first 24 hours. Results are presented as fold difference compare to untreated cells at 24 hours. (C) FACS analysis of annexin V/7-AAD stained cells for determination of apoptosis and cell death. Single annexin V positive cells (x-axis, bottom right corner) represent cells undergoing apoptosis, and double positive cells (annexin V^+^ and 7-AAD^+^, right top population) represent dead cells. A slight increase of apoptotic and dead cells was detected at 12 hours which progressed to significant after 24 hours of induction. (D) ATP assay on the cells induced for 24 hours demonstrated that antioxidants (AsAc: ascorbic acid (21.25 mM), B-MET: β-mercaptoethanol (0.5 mM), MTG: monothioglycerol (2.25 mM)) did not have any beneficial effect on cell viability even in cells treated with the low dose of doxycycline (32 ng/ml). (E) Morphology of cells, either uninduced (Control), mDUX-induced (Dox, 125 mg/ml) or induced and treated with antioxidants. (F) Morphology of mDUX inducible fibroblasts (i3T3-mDUX) and inducible mDUX embryonic stem cells (iES-mDUX) (G) after 24 hours of induction with 500 ng/ml doxycyline. mDUX expressed at high levels induces cell death in fibroblasts and embryonic stem cells. (H) ATP assay for effects of doxycycline on viability of control C2C12 and iC2C12 cells after 48 hours of treatment. Results are presented as fold difference compare to untreated C2C12 cells.

mDUX expressed at high level in iC2C12-mDUX myoblasts induced rapid cell death within 24 hours (Fig. 1A). Detached cell aggregates floating in the culture medium were visible after 18 hours of induction. Using propidium iodide staining and FACS analyses we confirmed that floating cells are dead and not live but detached cells (data not shown). Remarkably, we did not observe significant structural or morphological changes in the induced cells prior to lifting and dying (Fig. 1A) as we have observed with DUX4 which, before dying, stretch out to acquire an elongated cell shape and ovoid nucleus [14]. Necrotic phenotypes have been observed in myoblasts cultured from FSHD patients [18]. We next used an ATP assay to analyze viability of the mDUX expressing myoblasts over a wide range of levels of induction with doxycycline. A significant decrease of cell viability was detected in the cultures induced with as little as 32 ng/mL doxycyline in the first 24 hours (Fig. 1B). This trend increased in the following 24 hours, where toxicity became obvious even in the cells induced with lower doses (16 ng/mL). We did not detect any significant effect of doxycycline on the parental iC2C12 nor C2C12 (grand-parental) cells (Figure 1H). By using annexin V/7-AAD staining we demonstrated that the cells undergo apoptosis and did not just decrease the proliferation rate (Figure 1C). At high concentrations of doxycycline (500 ng/mL), the first signs of increased apoptosis and cell death were evident after 12 hours of induction. By 24 hours, 30% of cell-sized events were apoptotic and 44% were dead (Fig. 1C). This data demonstrates a clear deleterious effect of mDUX on myoblasts, very much like that exhibited by DUX4 when expressed at high levels [12], [14]. FSHD myoblasts have been reported to be highly susceptibile to oxidative stress [18] and several studies analyzing RNA and protein expression in FSHD biopsy samples confirmed misregulation of a number of factors involved in glutathione redox buffering [16], [18], [22]. We observed similar effects when DUX4 was expressed in iC2C12-DUX4 cells and showed that antioxidants were able to moderate toxicity of even very high levels of DUX4 expression [14]. To test whether antioxidants could rescue the toxic phenotypes of mDUX, we treated iC2C12-mDUX cells with ascorbic acid, β-mercaptoethanol and monothioglycerol. iC2C12-mDUX were induced with 32 and 125 ng/mL doxycyline, treated with serial dilutions of these antioxidants and analyzed by ATP assay after 24 hours. Surprisingly, we did not observe any beneficial effect of the antioxidants even in the cells which were induced with low levels of doxycyline (32 ng/mL) (Fig. 1D and E), demonstrating that key aspects of mDUX toxicity are distinct or perhaps more potent that those of DUX4.

To determine whether mDUX was exclusively toxic for myoblasts or would be toxic in other cell types, we evaluated fibroblasts (i3T3-mDUX) [14] and murine embryonic stem cells (iES-mDUX) [23]. mDUX expressed at high levels in fibroblasts and ES cells also induced rapid cell death (Fig. 1F and G). Together these results show that mDUX, like human DUX4, is generally toxic for various cell types when expressed at high levels. However, unlike DUX4, oxidative stress is not the principal reason for death of cells expressing mDUX. Given this toxicity, it is remarkable that transcription of mDUX has been reported in various tissues, including robust expression in CNS, lung and liver [20]. Which cell types within those organs, and at which stages they express mDUX, was not shown, however splicing variants were suggested, and these could theoretically result in non-toxic versions of the protein. We used the whole mDUX ORF sequence (2 kb) as a template to generate *in situ* hybridization probes and were not able to detect any robust and convincing mDUX expression (from the sense strand) in embryonic neural tube, adult brain or muscle tissue (data not shown).

### mDUX and myogenic regulators

**Figure 2:**
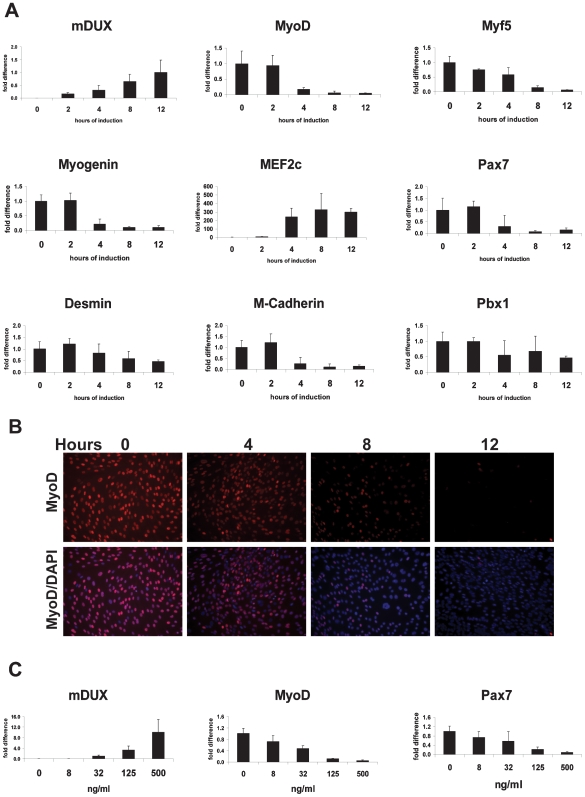
mDUX and myogenic regulators. qRT-PCR for mDUX and myogenic genes in iC2C12-mDUX cells evaluated at different times (A, using 500 ng/mL doxycycline) or doses (C, at 12 hours). Results are presented as fold difference compared to uninduced cells (0 ng/ml) except for the expression of mDUX in which 12 hours of induction was taken as the group for comparison. Error bars represent the STDEV. Induction with 8 ng/mL of doxycycline was sufficient for significant down-regulation of MyoD. (B) Immunofluorescence for detection of MyoD (red) in iC2C12-mDUX cells induced during the time course of 12 hours. Nuclei were stained with DAPI (blue). A notable decrease in the number of the positive-staining nuclei and the intensity of the staining was detected as early as 4 hours after induction. (C) Expression of mDUX, MyoD, and Pax7 when mDUX is induced with various concentrations of doxycycline.

mDUX, DUX4 and DUX4c each contain two homeoboxes of the Pax family, each with significant sequence similarity to the Pax3 and Pax7 homeoboxes [14]. These Pax factors are the master myogenic regulators of embryonic and adult myogenesis, respectively (for review see [24]). In our previous work, we demonstrated that DUX4 and DUX4c both interfere with the expression of myogenic regulators and suggested that this was due to competitive binding between DUX and Pax proteins for the same regulatory sites in these genes. The apical targets of Pax3/Pax7 in the cascade of myogenesis are MyoD and Myf5 [25], [26]. One interesting difference between DUX4 and DUX4c in this regard is that DUX4 upregulates Myf5 about two-fold in myoblasts (more in fibroblasts) whereas DUX4c represses Myf5 [13], [14]. For this reason we analyzed the expression of MyoD and Myf5 in mDUX-expressing myoblasts. As predicted, transcription of MyoD was rapidly downregulated (seen by 4 hours post-induction) with 500 ng/mL doxycyline (Fig. 2A). We confirmed these rapid kinetics at the protein level using immunofluorescence (Fig. 2B). For Myf5 we detected a slight downregulation after 4 hours and a more significant downregulation after 8 hours of induction (Fig. 2A). As a consequence of the MyoD suppression, some of its target genes including myogenin and m-cadherin were also downregulated (Fig. 2A). MyoD binds to noncanonical E boxes in the myogenin gene through interactions with Pbx1 and Meis1 [27]. In mDUX-induced cells, we detected a slight repression of Pbx1 (Fig. 2A). On the other hand Pbx3, Pbx4, Meis1 and Meis2 remained unchanged (data not shown). Interestingly, we found that Pax7 was also suppressed, suggesting the possibility of interference with a Pax7 positive autoregulatory loop. Three lines of evidence suggest that these changes are not simply due to cells becoming apoptotic and downregulating gene expression generally. First, DUX4 has many upregulated targets [14], and we discovered at least one upregulated target of mDUX, namely MEF2C (Fig. 2A). Second, genes downstream of MyoD are also repressed, but with correspondingly delayed kinetics. For example, desmin expression is not significantly affected until 12 hours (Fig. 2A), suggesting a secondary effect resulting from MyoD depletion. Third, MyoD was downregulated by mDUX induced with as little as 8 ng/ml doxycyline for 24 hours (Fig. 2C). At that level we did not observe any significant toxic effect even after 48 hours of induction. MyoD and Myf5 are critical myogenic factors. In order for progenitors to be able to undergo myogenic differentiation, they must at one point express MyoD and/or Myf5; accordingly, mice null for both MyoD and Myf5 lack all skeletal muscles [28]. MyoD has been suggested to be a factor in the pathogenesis of the FSHD by studies demonstrating misregulation of MyoD and its target genes in myoblasts from the patients [15], [16].

### mDUX and myogenic differentiation

**Figure 3:**
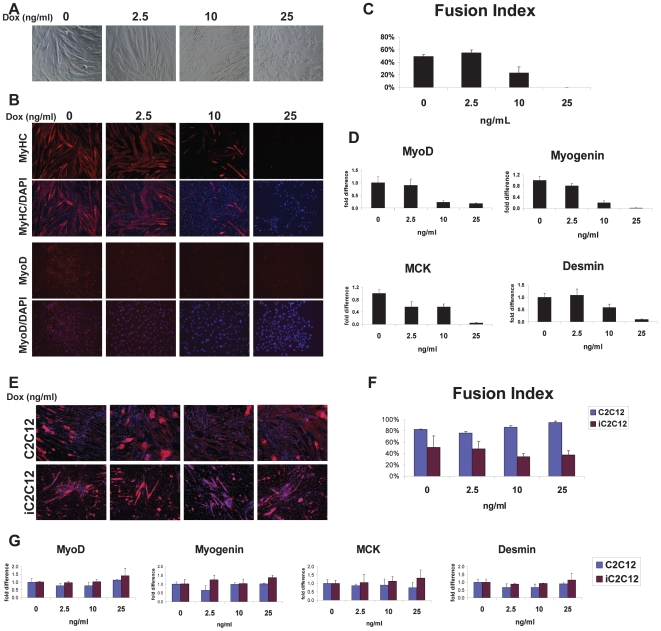
mDUX and myogenic differentiation. (A) Phase-contrast microscopy of iC2C12-mDUX cells induced with doxycyline through 6 days of differentiation. (B) Immunofluorescence for detection of MyHC (red, upper panels) and MyoD (red, lower panels) in cells induced with low levels of doxycycline. Nuclei were counterstained with DAPI (blue). iC2C12-mDUX cells were in differentiation medium for 6 days when myotube fusion index was calculated (C). Significantly diminished myogenic differentiation was observed in the cells induced with 10 ng/mL doxycyline. (D) Inhibition of differentiation was confirmed by qRT-PCR. Results are presented as fold difference compared to uninduced cells (0 ng/mL) and the error bars represent the STDEV. (E) Immunofluorescence for detection of MyHC (red) in C2C12 and iC2C12 control cells after 6 days of differentiation and treatment with different concentrations of doxycycline. (F) Calculated fusion index and (G) gene expression analyses of differentiated C2C12 and iC2C12 control cells. Doxycycline by itself did not have any significant effect on myoblast (C2C12 and iC2C12) differentiation.

To evaluate whether downregulation of MyoD and it target genes lead to the functional abnormalities, we analyzed the potential of mDUX expressing myoblasts to fuse and form myotubes. Differentiation was induced by culturing confluent iC2C12-mDUX cells in nutrition/growth factor-poor medium (DMEM supplemented with 2% horse serum). During the course of differentiation, mDUX was induced with 2.5, 10 and 25 ng/ml of doxycyline. In the presence of 10 or 25 ng/ml doxycycline, differentiation was visibly impaired, while non-treated cells fused and formed typical elongated myotubes (Fig. 3A). At the terminal stages of the experiment (after day 6 of differentiation) some dead cells were observed at 10 and 25 ng/mL dox, but the majority were alive and still attached to the plate, and at 25 ng/mL, even sporadic myotubes were not seen (Fig. 3A). Differentiation and formation of mature myotubes was confirmed by immunofluorescence staining for MyHC (Fig. 3B) and measuring the myotube fusion index (percent of nuclei within myotubes). The fusion index in the control and 2.5 ng/mL-induced cells was slightly over 50%. However the number of the nuclei within myotubes in the 10 ng/mL-induced group was much decreased, and the myotubes that did form were smaller and shorter. Immunostaining and qRT-PCR for MyoD revealed reduced expression in the induced cells which was proportional to the levels of inhibition of differentiation (Fig. 3B and D). Gene expression analyses of markers of differentiation, myogenin, MCK and desmin further confirmed diminished differentiation in the mDUX-induced cells (Fig. 3D). To eliminate the influence of doxycyline by itself on myogenic differentiation we conducted the same experiments on the iC2C12 parental and C2C12 (grand-parental) cell lines. We did not find any significant doxycyline-related inhibition of differentiation by immunofluorescence for MyHC, calculation of myotube fusion index, or analysis of gene expression (Figure 3E, F and G) [13], [14]. Since mDUX inhibited differentiation at non-toxic levels of induction, we assume that this is due to interference with myogenic pathways. In support of this explanation, other non-toxic versions of DUX4, for example DUX4c [13] or certain mutations in the c-terminus of DUX4 (our unpublished data) display potent inhibition of differentiation as well as inhibition of MyoD and Myf5 expression. It was previously reported that increased numbers of D4Z4 repeats transfected into C2C12 myoblasts impairs their differentiation ability [29]. Furthermore, morphological differentiation abnormalities were reported in myoblasts and mesangioblasts isolated from FSHD patients [17], [30]. The ability of low levels of mDUX to interfere with the differentiation of myoblasts, together with its toxicity at high levels, suggests that its mechanism of action is conserved with DUX4 and relevant to FSHD.

### *mDUX* expression in *Xenopus laevis*

**Figure 4:**
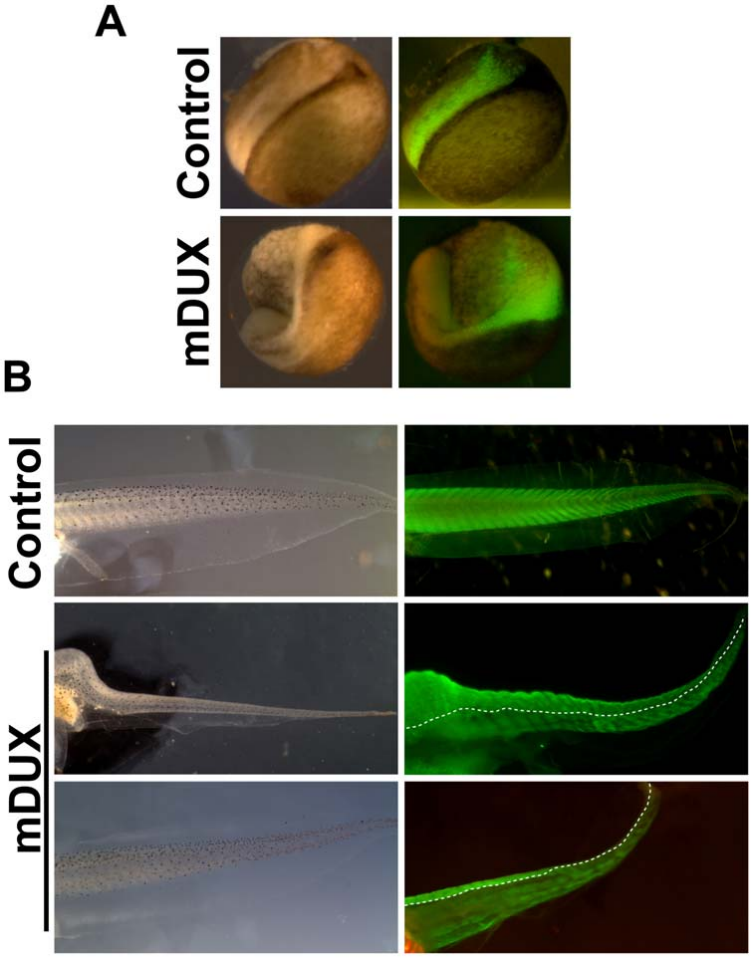
mDUX expression in Xenopus. (A) Neurula stage embryos following injection of *GFP* mRNA alone (control) or *GFP+mDUX* mRNA. The green color indicates the domain filled by the RNA injection. The control has a normal neural plate while the mDUX case is very abnormal due to deranged gastrulation movements. (B) Tail muscle pattern in stage 45 tadpoles. The green color shows immunostaining with 12/101 antibody. Control shows the normal pattern of myotomes. Two examples (mDUX) demonstrate how injection into blastomeres V2.1 and 2.2 results in an inhibition of muscle differentiation on the injected (left) side.

Previous results on the *in vitro* effect of DUX4 and the data presented in this paper suggest that mDUX would interfere with myogenesis *in vivo*. Therefore we tested the effects of *in vivo* expression of mDUX in a whole animal model: embryos of the frog *Xenopus laevis*. Embryos were microinjected with *mDUX plus GFP* (1 ng+100 pg) or *GFP* (1 ng) mRNA alone at the four-cell or sixteen-cell stage (NF) embryos. At the four-cell stage, *mDUX* (n = 120) or *GFP* (n = 120) mRNA was injected into one blastomere of the dorsal animal side and the embryos were allowed to develop under normal conditions at room temperature. They were observed under a dissecting microscope at 1, 3, and 7 days post injection and those embryos expressing mDUX or control RNA were identified by positive GFP fluorescence and were separated for further analysis. 89% of embryos expressing mDUX (GFP^+^) were observed to have gastrulation defects compared to only 7% of GFP control injected embryos one day post injection (Fig. 4A). All embryos expressing mDUX died prior to day 7 (stage 45) showing severe defects in morphology consistent with the initial defects in gastrulation. No large scale cell death was apparent, which suggests that at this concentration, the main deleterious effect of mDUX is to interfere with cell and tissue movements, in addition to any effects it may have on myogenesis. To bypass the effects of early mDUX expression on gastrulation movements, we then performed injections into blastomeres V2.1 and V2.2 on one side of a sixteen-cell stage embryo. Previously described cell fate maps have shown these blastomeres to contribute primarily to cells of the dorsal and ventral somite and the corresponding musculature of the *Xenopus* tadpole tail [31], [32]. *mDUX* (n = 120) embryos were observed to develop normally through gastrulation one day post-injection but had significant defects in tail development at three days post-injection compared to GFP controls (n = 120). At seven days (stage 45, NF) 72% of mDUX tadpoles had truncated or reduced tails compared to only 6% of controls. Whole mount immunostaining of tadpoles with 12/101 antibody, which identifies skeletal muscle, showed a delay in myogenic differentiation and a decrease in the number of muscle fibers in mDUX tadpoles on the injected side, compared to the contralateral and uninjected controls (Fig. 4B). Together these results are consistent with mDUX having an inhibitory effect on muscle differentiation, but also showing significant other effects manifested by the derangement of gastrulation movements.

### Competition with Pax factors

**Figure 5:**
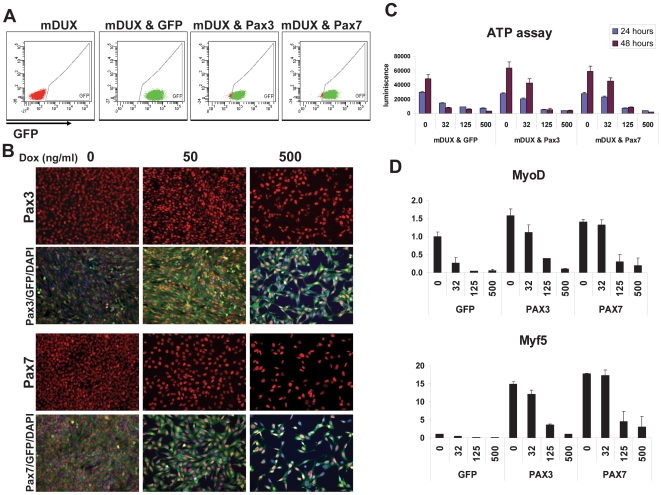
Pax3 and Pax7 compete with mDUX. (A) FACS analysis of iC2C12-mDUX cells transduced with MSCV retroviral constructs caring GFP, Pax3-ires-GFP or Pax7-ires-GFP. Almost all of the cells at the time of the experiment stably express GFP (x-axis). (B) Immunofluorescence for Pax3 or Pax7 (red) and GFP (green) reveals that Pax3 and Pax7 are expressed in GFP^+^ cells. Cell number is decreased in induced samples due to toxicity of mDUX. (C) ATP assay for determination of cell viability in iC2C12-mDUX cells transduced with MSCV-ires-GFP, MSCV-Pax3-ires-GFP or MSCV-Pax7-ires-GFP. Cells were induced with various concentrations of doxycycline for 24 and 48 hours. Pax3- and Pax7-expressing cells are largely resistant to the toxicity of mDUX induced by 32 ng/mL dox even after 48 hours of induction. (D) qRT-PCR analyses for MyoD and Myf5 in the cells shown in (C), induced for 18 hours. Expression of MyoD and Myf5 is strongly repressed at 32 ng/mL induction in the control cells, but not the Pax3 or Pax7 expressing cells.

The DUX4 homeodomain showed very high similarity to the homeodomains of Pax3 and Pax7 [14]. This similarity was the key factor in our hypothesis that DUX proteins, including mDUX, interfere with Pax3 and Pax7 by competing for the same DNA targets. One prediction of this hypothesis is that excess Pax3 or Pax7 in cells should block or reduce the toxicity of DUX proteins, and we found this to be the case for DUX4 [14]. We therefore transduced iC2C12-mDUX cells with Pax3 or Pax7 using retroviral vectors bearing ires-GFP reporters (allowing identification of transduced cells) or GFP alone. Transduced cells (60–70% infection rate) were FACS-sorted to obtain a homogeneous cell population. Subsequent FACS analyses confirmed that almost all of the sorted, expanded cells were GFP^+^ (Fig. 5A) and immunofluorescent staining confirmed that the GFP^+^ cells expressed Pax3 or Pax7 (Fig. 5B). To evaluate the potential competitive interaction, we induced mDUX at different levels and cell viability was followed over 48 hours. This experiment demonstrated that cells overexpressing Pax3 or Pax7 are resistant to the toxicity of mDUX induced by 32 ng/mL (Fig. 3C). The rescue was complete in the first 24 hours and still significant after 48 hours. Importantly, within 48 hours, the induced cells (32 ng/mL) did not merely survive latently, but proliferated as indicated by a higher ATP content at 48 hours compared to 24 hours (Fig. 5C). On the other hand, cell death was rapid in the control cells expressing only GFP. The competitive effect of Pax3 and Pax7 was also evident on the expression of myogenic regulators. MyoD and its target genes, which were rapidly downregulated by mDUX even with low levels of doxycyline (Fig. 2), were resistant to low levels (32 ng/ml) of mDUX in the Pax3 or Pax7 transduced populations but not in the GFP-only controls (Fig. 4D). This interaction was indeed competitive, because at higher levels of induction, the mDUX repressive effect dominated. This competition reveals that mDUX and DUX4 act in similar mechanistic pathways.

### Conclusions

In this study we show that mDUX provokes myopathic molecular and physiological cellular changes related to those caused by human DUX4. Specific differences between mDUX and DUX4 were noted: mDUX downregulated Myf5 whereas DUX4 upregulated Myf5, mDUX-expressing cells did not stretch before dying whereas DUX4-expressing cells did, and mDUX-mediated death could not be rescued by antioxidants, as it could to a significant degree for DUX4. With regard to Myf5, mDUX actually behaves as DUX4c, which downregulated both MyoD and Myf5. With regard to cellular morphology and oxidative stress, it should be noted that DUX4 provokes multiple deleterious pathways, and although antioxidants could fully rescue from low levels of DUX4, it could only partially protect against high levels of DUX4, and then only for a few days. The distinction between mDUX and DUX4 in this regard may be more a distinction of the degree to which various similar pathways are affected, rather than a qualitatively different mechanism of cell death. Like DUX4, mDUX shows a biphasic response: high levels were toxic while low levels perturbed differentiation and myogenic gene expression. Considering the toxic effects of mDUX expression, its interaction with myogenic regulators and inhibition of myogenic differentiation in both cell- and organism-based assays, we propose that the mouse D4Z4-like arrays represent a genetic unit functionally orthologous to the DUX4-bearing repeats at human 4q35.2. Although the normal function of these repeats remains unknown, the abnormal expression of the double homeodomain protein they encode results in clear myopathic effects, with several lines of evidence suggesting that these effects are relevant to FSHD. We therefore propose that intervention in the normal expression or repression of mDUX through either deleting integral numbers of murine repeats, insertion of a strong enhancer into the vicinity of the repeat array, or placing sequences from the repeat array, for example the mDUX ORF, under the control of a tissue-specific, regulated promoter would represent physiologically relevant ways to model FSHD in mice.

Because the mechanism linking the D4Z4 deletion to myopathy has not been established, there is an urgent need for animal models that incorporate D4Z4 sequences. We have developed a model based on expression of the mDUX ORF during *Xenopus* development. This model makes evident the myopathic nature of D4Z4 itself. Furthermore, it is experimentally tractable and amenable to a medium-throughput approach. It represents an example of a vertebrate model based on sequences encoded by the repeats affected by the FSHD deletion itself, and highlights the utility of using rapidly developing lower vertebrates to model human genetic disease.

## Materials and Methods

### Cloning of mDUX

The mDUX ORF was amplified from 100 ng mouse genomic DNA by 0.4 uM forward (atggcagaagctggcag) and 0.4 uM reverse (tcagagcatatctagaagagtct) specific primers using FastStart High Fidelity PCR mixture ( 5 ul 10× buffer with 1.8 mM MgCl_2_ , 200 uM of each dNTP and 2% DMSO; all from Roche). Amplification was done with 35 cycles, each consist of 30 s denaturation at 95°C, anneling for 30 s at 60°C and elongation for 140 s at 72°C. Sequencing confirmed that the PCR product corresponded to mDUX clone C1 (accession No. AM398151). The PCR products were subcloned into p2lox [23] and used for gene targeting.

### Cell culture

C2C12 cells and NIH 3T3 fibroblasts were cultured in high glucose Dulbecco's Modified Eagle Media (DMEM) with L-glutamine and sodium pyruvate (Gibco), penicillin and streptomycin (P/S, Gibco) and 10% fetal bovine serum (FBS, HyClone) at 37°C in 5% O_2_/5% CO_2_. For myotube formation, C2C12 cells were cultured on gelatin-coated plates until confluence, and then washed with serum-free DMEM and differentiated with DMEM supplemented with 2% horse serum (HS, Invitrogen) and insulin (Sigma) for 4–6 days. Mouse embryonic stem (ES) cells were cultured on irradiated MEFs in “Knockout” DMEM/15% FBS, P/S, glutamine, nonessential amino acids, 0.1 mM β-mercaptoethanol, and 100 U/ml LIF (Peprotech). iC2C12-mDUX, i3T3-mDUX and iES-mDUX were generated as previously we described [14], [23].

### Retro-virus production and generation of Pax3/Pax7-expressing iC2C12-mDUX cell lines

Retroviral supernatant was produced in 293T cells cultured in DMEM/10% FBS. Retroviral constructs (1 ug of pMSCV-ires-GFP to generate pMSCV-Pax3-ires-GFP and pMSCV-Pax7-ires-GFP [14]) were co-transfected with 1 ug pCL-Eco packaging constructs using Fugine 6 (6 ul, Roche). Medium was changed after 24 hours and the viral supernatant was collected at 48 hours post-transfection. Filtered supernatant (0.45 µm) was applied to the cells with polybrene (4 µg/mL). Spin-infection was performed at 2000 g at 33°C for 90 min. Cells were incubated overnight at 37°C in 5% O_2_/5% CO_2_ after which the supernatant was replaced with fresh medium. Several days after infection iC2C12-mDUX cells were trypsinized and analyzed for GFP expression using a FACS Aria (BD). GFP^+^ cells were sorted to obtain ∼100% positive expressing cell lines using FACS Aria (BD).

### ATP assay

Cells were plated in a 96 well plate (1200 cells/well). The genes were induced the following day with various doxycycline concentrations for 24 or 48 hours. To assay ATP levels, medium from the wells was removed and cells were lysed by 50 ul ATPlite for 1 minute (ATP determination Kit, Molecular Probe, Eugene, OR). The intensity of luminescence was assayed on a POLARstar Optima Microplate Reader (BMG LABTECH, Offenburg, Germany). Data is presented as fold difference compare to the control and the error bars represent SDEV (n = 8).

### Immunofluorescence

Cells were cultured on gelatin coated 24 well plates, fixed with 4% paraformaldehyde for 20 min, permeablized by 0.3%Triton X-100 for 30 min and blocked by 3% BSA for 1 hour at room temperature. The cells were incubated with the primary antibodies (mouse monoclonal anti mouse MyoD (1∶250, BD Biosciences), mouse monoclonal anti-Pax3 and anti-Pax7 (1∶250, R&D Systems), mouse anti-myosin heavy chain (1∶20, Developmental Studies Hybridoma Bank, U Iowa), polyclonal chicken anti-GFP antibody (1∶500, AbCam) diluted in PBS/3% BSA at 4°C overnight. The secondary antibodies Alexa Fluor 488 and/or 555 (1∶500, Invitrogen) were applied at room temperature for 45 min. Cells were counterstained using 4′,6-diamidino-2-phenylindole (DAPI, Invitrogen) to visualize nuclei.

**Whole mount immunohistochemistry** was carried out as described previously [33]. Briefly, stage 46 (NF) tadpoles were fixed in Zamboni's fixative (40 mM NaH2PO4, 120 mM Na2HPO4, 2% PFA, 0.1% saturated picric acid) overnight at 4°C. Tadpoles were washed in PBSA three times for 10 minutes each and bleached in 5% (w/v) H_2_O_2_ (Sigma) in PBSA for 1 hour under direct light. Samples were washed in BBT (PBSA+1% (w/v) BSA+0.1% (w/v) Triton X-100), blocked in BBT containing 5% horse serum for 1 hour and incubated overnight at 4°C in blocking solution containing a mouse monoclonal antibody 12/101 (1∶100; grown in the lab, original gift from Liz Jones). Tadpoles were incubated overnight at 4°C in BBT with 5% horse serum containing Alexa Fluor 488 anti-mouse IgG (1∶200; Invitrogen) labeled secondary antibody. Tadpoles were visualized using a Leica Fluo III fluorescent dissecting microscope with a GFP2 filter set and photographed with Spot RT Camera (Diagnostic instruments).

### Quantitative Real Time RT-PCR (qRT-PCR)

Total RNA was extracted with Trizol (Invitrogen) and cDNA was generated using 1 µg DNase treated RNA with oligo-dT primer and TermoScript (Invitrogen) following the manufacture instruction. Quantitative PCR was performed by using pre-made primers/probes (GAPDH Mm99999915_g1, MyoD1 Mm00440387_m1, Myf5Mm00435125_m1, myogenin Mm00446194_m1, desmin Mm00802455_m1 and MCK Mm00432556_m1 all from Applied Biosystems) and TaqMan PCR premixture on 7500 real time PCR System (Applied Biosystems). Glyceraldehyde phosphate dehydrogenase (GAPDH) was used as the internal standard. For detection of mDUX, SyberGeeen (Applied Biosystems) premixture and the following f: gcactcaagcagacagcaca and r: gtgtccatttcgtcccatgt primers were used. Gene expression was normalized and analyzed using the delta Ct method by 7500 System Software (Applied Biosystems). Results were presented as fold difference of the mean compared to the control (ΔΔCt). All reactions were performed at least in triplicate and the error bar is STDEV.

### Annexin V Staining

iC2C12-mDUX cultured in 6 well plates were induced with 500 ng/ml dox for 4, 8, 12 and 24 hours. **Annexin V/7-AAD stainig was done using Staining** BD Bioscience kit and following the manufacturer's instructions. In brief, from each group 10^5^ trypsin-detached cells were stained with APC-coupled Annexin V antibody and 7-AAD dye in binding buffer (BD Bioscience). Dead and apoptotic cells were detected by FACS analyses.

### Embryos, tadpoles and microinjection

Animal care was provided in accordance with NIH guidelines with oversight provided by the University of Minnesota Animal Care and Use Committee. *Xenopus laevis* embryos were obtained by *in vitro* fertilization and staged according to the Nieuwkoop and Faber (NF) tables [34]. Embryos were dejelled with 2% cysteine (Sigma) (pH 7.8) and then cultured in 0.1X Normal Amphibian Medium (NAM; [35]) for approximately 5 days to NF stage 46/47. RNA used for microinjections was transcribed *in vitro* and capped using mMessage Machine (Ambion) from plasmids containing either the mouse DUX4 CDS or nuclear GFP (pcDNA 3.1 NucGFP) and was injected into early *Xenopus laevis* embryos using a Nanoject injector (Drummond Scientific company). *mDUX* (1 ng mDUX+100 pg nucGFP) or *nucGFP* (1 ng nuclear GFP) mRNA was injected in a volume of 2.3 nl into either one blastomere of dorsal animal side at the four-cell stage or into V2.1 and 2.2 blastomeres of at the sixteen-cell stage [32]. The embryos were incubated in half strength NAM (NAM/2)+3% Ficoll during injection and kept in this medium for 2–3 hours post injection before being transferred to NAM/10 solution until staining.

### Statistical analyses

All experiments were done at least 3 times. Data shown for Real Time PCR are the mean±STDEV. Difference between means was compare by the two-tailed Student test and was considered significantly different at P<0.05.
